# Study of Low Terahertz Radar Signal Backscattering for Surface Identification

**DOI:** 10.3390/s21092954

**Published:** 2021-04-23

**Authors:** Shahrzad Minooee Sabery, Aleksandr Bystrov, Miguel Navarro-Cía, Peter Gardner, Marina Gashinova

**Affiliations:** 1 School of Engineering, University of Birmingham, Birmingham B15 2TT, UK; SXM1147@student.bham.ac.uk (S.M.S.); m.navarro-cia@bham.ac.uk (M.N.-C.); p.gardner@bham.ac.uk (P.G.); m.s.gashinova@bham.ac.uk (M.G.); 2 School of Physics and Astronomy, University of Birmingham, Birmingham B15 2TT, UK

**Keywords:** surface roughness, low terahertz, radar cross-section, remote sensing, electromagnetic scattering, time-domain spectrometer

## Abstract

This study explores the scattering of signals within the mm and low Terahertz frequency range, represented by frequencies 79 GHz, 150 GHz, 300 GHz, and 670 GHz, from surfaces with different roughness, to demonstrate advantages of low THz radar for surface discrimination for automotive sensing. The responses of four test surfaces of different roughness were measured and their normalized radar cross sections were estimated as a function of grazing angle and polarization. The Fraunhofer criterion was used as a guideline for determining the type of backscattering (specular and diffuse). The proposed experimental technique provides high accuracy of backscattering coefficient measurement depending on the frequency of the signal, polarization, and grazing angle. An empirical scattering model was used to provide a reference. To compare theoretical and experimental results of the signal scattering on test surfaces, the permittivity of sandpaper has been measured using time-domain spectroscopy. It was shown that the empirical methods for diffuse radar signal scattering developed for lower radar frequencies can be extended for the low THz range with sufficient accuracy. The results obtained will provide reference information for creating remote surface identification systems for automotive use, which will be of particular advantage in surface classification, object classification, and path determination in autonomous automotive vehicle operation.

## 1. Introduction

In recent years, radars operating in the low THz range, from 0.1 THz to 1 THz, have been developed for a wide range of applications such as standoff personnel screening [1], material characterization [2] and, importantly, have been proposed as a candidate radar to provide high-resolution imagery for future autonomy [3,4,5]. The use of such radars allows an autonomous vehicle to effectively classify road objects and perform route planning [6,7]. Because of the wide operational bandwidth available in low THz radar [8,9], compared to current ISM standard automotive radars, significantly higher range resolution is achievable, leading to more image-like scene characterization by such a radar. At the same time, unlike LIDAR and optical sensors, low THz sensors have the advantage of robust operation in harsh weather and lighting conditions [10,11,12]. Importantly, operation at higher frequencies leads to more compact electronics components, in particular antennas, thereby responding to challenges of more and more dense packaging of multiple sensors and processing systems for modern and future cars. Until recently, the high cost was an obstacle to the widespread implementation of low THz radar systems, but with the development of appropriate technologies [13], it has been steadily decreasing.

The atmospheric attenuation, primarily due to water vapor absorption, is significant in many spectral regions in the low THz band as shown in [14], especially in adverse weather conditions [11,15]. However, there are transmission windows around 340 GHz, 400 GHz, and 650 GHz where atmospheric loss in clear air does not exceed 10 dB/km, 20 dB/km, and 60 dB/km, respectively [14]. The results obtained in [16] for frequencies of 77 GHz and 300 GHz show that even in heavy rain the attenuation of the radar signal did not exceed 20 dB/km, and in [17] measured attenuation during snowfall was below 15 dB/km. Therefore, for the automotive radar operational range of up to 100 m, atmospheric loss will not exceed 10 dB and makes a relatively small contribution to the power budget needed to guarantee system efficiency [18]. The road surface classification is an important outstanding problem in the implementation of autonomous vehicles. The extensive review of the papers on surface identification (Surface ID) using radar techniques can be found in [19]. As follows from the review, the most promising method for solving this problem is the analysis of the features of the backscattered signal with different polarization [20].

The principles of Surface ID are based on the theory of electromagnetic signal reflection from surfaces. Surface scattering is a function of surface roughness, characterized by its statistical parameters such as rms height and correlation length, radar frequency, grazing angle, and the effective permittivity of the material of the surface [21]. The problem of signal scattering from random surfaces has been investigated for many years [22]. There are two well-known solutions accounting for different ratios of wavelength and surface heights: (i) the Kirchhoff approximation, when surface roughness is larger or comparable to the wavelength and (ii) the small perturbation model, which performs better if the variation of surface heights is small relative to the wavelength [23]. Several models have been developed that combine these two, but they all have limitations on the signal frequency, on the surface dielectric constant and roughness characteristics [24]. Various empirical approaches are also widely used in the analysis of backscattering, where theoretical models are expanded or modified based on empirical observations to improve the performance of the original model in the interpretation of experimental data [25,26].

Advances in modern electronics have led to the emergence of commercially available radar components with frequencies above 100 GHz and this frequency range is currently of particular interest to automotive radar developers. At the same time, in most publications the analysis of radar signal backscattering is carried out in the frequency range up to 100 GHz and there is a limited number of studies investigating scattering of signals above 200 GHz. Most of these publications present research on diffuse scattering from rough surfaces in THz communication channels using time-domain spectrometers [27,28]. These works cover bistatic scattering measurements in the frequency range from 100 GHz to 1 THz from a set of specially manufactured scattering targets with known roughness parameters. In [29] surface scattering was measured using a vector network analyzer (VNA) for 325–500 GHz and a dedicated system for 650 GHz. Of particular interest is [30], which examines 222 GHz polarimetric monostatic radar backscatter response of different types of real surfaces, such as asphalt, concrete, dirt, and grass of various length.

The lack of comprehensive and consistent surface scattering studies over both the millimeter-wave and THz range simultaneously motivates this paper. Hence, the aim here is to study and characterize the effect of signal backscattering from rough surfaces in the range of millimeter-wave and low THz frequencies (79–670 GHz) within the context of Surface ID for automotive sensing. The groundwork reported here for sandpapers with known geometrical parameters identifies the impact of all critical parameters (e.g., surface roughness and dielectric properties, grazing angle, and polarization) on the surface backscattering and lays the foundations to make predictions for real road surfaces. Preliminary results related to this study were presented in [31]. In this paper, we propose an improved experimental technique for measuring normalized radar cross-section (RCS) of rough surfaces and present a method to calculate the normalized RCS of surfaces of different roughness as a function of radar and topology parameters, such as polarization and aspect angle. The obtained measurement results were analyzed for compliance with the Fraunhofer criterion and were compared with normalized RCS, calculated based on an improved empirical model.

This paper is structured as follows. In Section 2, the type of signal scattering based on the Fraunhofer criterion is discussed. In Section 3, the methodology of backscattering coefficient measurement is described, the empirical model of signal scattering is discussed, and the results of sandpaper dielectric permittivity measurement are presented. The measured normalized RCS of sandpaper samples of four different levels of coarseness of the abrasive particles (grit) are presented in Section 4 and overall results are discussed. Finally, in Section 5, the conclusions and plans are formulated.

## 2. Backscattering of Radar Signal from Rough Surface

**Figure 1:**
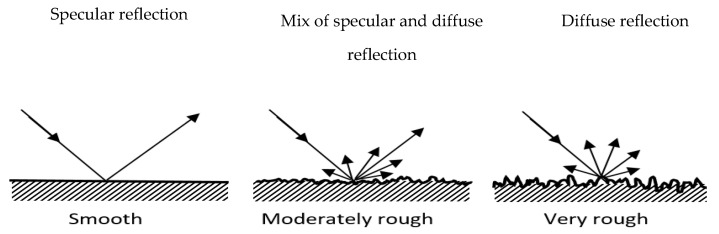
Reflection from smooth, moderately rough, and very rough surface.

To classify surfaces with different roughness using radar, we should understand the nature of the reflection of the signal from such surfaces. Two main mechanisms of signal scattering on surfaces with different roughness are shown in Figure 1. In a case of perfectly smooth surface only specular reflection will occur. When the surface becomes rougher, the ratio of specular reflection component will decrease and the diffuse reflections, re-radiating signal in all directions, will become more prominent. In a case of very rough surface, the diffuse reflection prevails over specular.

The type of scattering is defined by the surface rms height (root mean square average of the heights above or below a mean reference line) relative to wavelength. Based on the Fraunhofer criterion the surface is considered rough if the surface rms height *h* satisfies the inequality

(1) $$h\geq \frac{\lambda }{32cos\Theta }$$

where *Θ* is the angle of incidence relative to the surface normal and *λ* is the signal wavelength [32].

To evaluate the surface roughness relative to the wavelength, it is convenient to use electromagnetic roughness *kh*, where *k =* 2*π/λ* is the free space wave number. The Fraunhofer criterion (Equation (1)) can be expressed in terms of electromagnetic roughness as

(2) kh≥0.2/cosΘ

This implies another definition for surface roughness [23], according to which a surface may be considered relatively smooth if its *kh <* 0.2 and very rough if its *kh ≥* 2.

**Figure 2:**
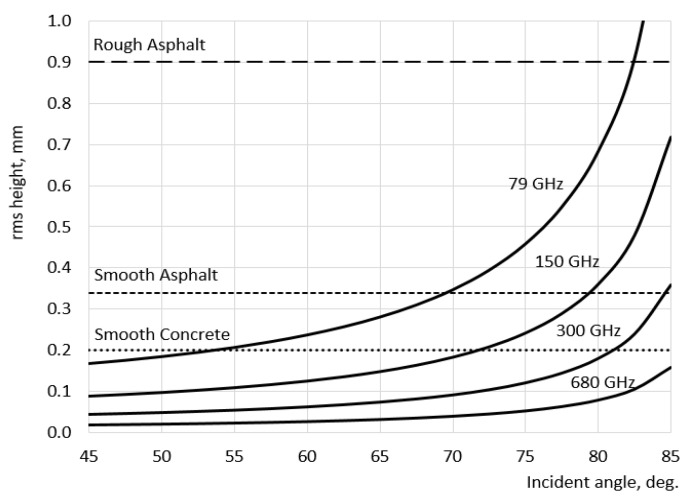
Roughness characterization of road surface as a function of incident angle and radar frequency [31].

Figure 2 shows graphs of rms height calculated in accordance with Equation (1) defining the surface as rough with respect to different radar frequencies. Diffuse scattering dominates in regions above lines for each frequency, ensuring non-zero return to radar receiver as the surface will become rough. Threshold lines with a constant value of *h* correspond to three typical road surfaces: 0.2 mm for smooth concrete, 0.34 mm for smooth asphalt, and 0.9 mm for rough asphalt [26].

The returned radar signal is defined by the RCS of the surface, considered as a distributed target. As follows from the radar equation [33], the probability of target detection in noisy environment depends on its RCS. In the case we are considering, target detection means the ability to extract surface features from the backscattered signal. From a consideration of Figure 2, we can draw preliminary conclusions about the potential applicability of a radar operating at different frequencies for surface classification.

From Figure 2 it follows that a 79 GHz radar will not be able to distinguish smooth asphalt from smooth concrete at angles over 70° (the corresponding rms values are below the curve for 79 GHz, which suggests that they all appear effectively smooth). At the same time, 300 GHz radar can distinguish these surfaces to an angle of about 85°, and a 680 GHz radar is potentially able to distinguish smooth asphalt from smooth concrete up to an angle over 85°. Therefore, by increasing the radar frequency, the ability to identify the type of road surface at higher incidence angle *Θ* will improve. From here, we can make a conclusion about good prospects for using low THz radars for surface recognition.

**Figure 3:**
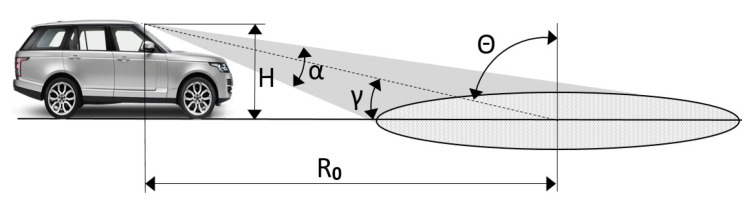
Automotive sensing scenario: *H*—Radar height over ground, *α*—Elevation beam width, *R*_0_—Ground range, *θ*—Incident angle, *γ*—Grazing angle.

In the case of automotive radars, low grazing (high incidence) angles are expected. Suppose we want to recognize a road surface at the distance of *R*_0_ = 10 m in front of the car (Figure 3). At a speed of 40 km/h, the car will cover such a distance in about a second. This is the time in which the automatic system must determine the optimum terrain response settings for the surface ahead to maintain momentum and vehicle control as one surface transitions to another. The maximum height at which the radar can be installed on a passenger car is approximately *H* = 1.5 m (e.g., mirror on the windshield). Under the conditions considered, the incident angle *Θ* will be approximately 81.5° which corresponds to a low grazing angle *γ* = 8.5°. If the radar is positioned within the bumper of the car at a height of 60 cm, the grazing angle will be only 3°.

In practical automotive radar implementation, at a small grazing angle, the illuminated surface footprint will extend over a wide ground range. Let us say that the elevation beam width *α* = 10°, then the illuminated area of the road would lie from 6 m to 24 m, which corresponds to the range of grazing angles from 13.5° to 3.5°. Therefore, when analyzing the backscattered signal, we must consider the backscattering at different grazing angles. Applying time gating, we can choose a strip of surface lying at a certain angle. Analysis of the dependence of the backscattered signal power on the grazing angle provides additional information about the properties of the surface.

## 3. Experimental Methodology

The focus of this research is on studying the low THz signal backscattering from surfaces with different roughness. To achieve this goal and attain accurate measurement, sandpaper of different coarseness (grit) was used as a reference surface. The results can easily be extended to the case of real road surfaces if their roughness and complex permittivity areas quantified. This section provides a concise and precise description of the experimental results, their interpretation, as well as the experimental conclusions that can be drawn.

### 3.1. Normalized RCS

The reflective properties of a surface are characterized by its normalized RCS. The normalized RCS of a distributed target is an ensemble average of the RCS *σ* per unit area:

(3) $$\sigma^{0}=\langle \sigma \rangle /A$$

where *A* is the illuminated area. In Section 3.4 we will show how this ratio can be determined using a VNA.

The return power when the target is illuminated by a monostatic radar can be calculated using the radar equation [33]:

(4) $$P_{R}=\frac{P_{T}G^{2}\lambda^{2}\sigma }{{\left(4\pi \right)}^{3}R_{0}^{4}}$$

where $P_{T}$ and $P_{R}$ are transmitted and received power, G is antenna gain, $R_{0}$ is the distance to the target, and *σ* is radar cross section. Thus, knowing the characteristics of the system, we can calculate *σ* from Equation (4).

This calculation can be simplified, and measurement accuracy improved, by calibrating the system and excluding the values of G and *λ* from the calculation. To calibrate the system, we carried out the free space measurement by placing the receiver at the distance 2 × *R*_0_ from the transmitter. Then the power received by the antenna is described by the Friis Transmission formula:

(5) $$P_{Rf}=\frac{P_{T}G^{2}\lambda^{2}}{{\left(4\pi \right)}^{2}4R_{0}^{2}}$$

From Equations (4) and (5) it follows that

(6) $$\sigma =\pi R_{0}^{2}\frac{P_{R}}{P_{Rf}}$$

The magnitude of the VNA transmission coefficient *S*_21_ is equal to the ratio of received power to transmitted power [34]

(7) $${\left|S_{21}\right|}^{2}=P_{R}/P_{T}$$

Therefore, RCS can be calculated as:

(8) $$\sigma =\pi R_{0}^{2}\frac{{\left|S_{21}\right|}^{2}}{{\left|S_{21f}\right|}^{2}}$$

where *S*_21*f*_ is the measured transmission coefficient in free space. Thus, by measuring S-parameters for the surface under test and in free space, and calculating the illuminated area, we can find the normalized RCS *σ*^0^ as defined in Equation (3).

### 3.2. Empirical Scattering Model

The empirical approaches to rough surface scattering are based on theoretical models and experimental observations. One of the best-known empirical models of radar backscattering response of natural surfaces was proposed in [25] for 0.1 < *kh* < 6.0 and 2.5 < *kl* < 20, where *l* is the correlation length; it was experimentally verified at 1.5–9.5 GHz. This model allows calculating co-polarization and cross-polarization ratios $\sigma_{HH}^{0}$/$\sigma_{VV}^{0}$ and $\sigma_{VH}^{0}$/$\sigma_{VV}^{0}$. Hereinafter, the first subscript indicates the transmitter antenna polarization, and the second subscript indicates the receiver antenna polarization.

According to this model, the co-polarized backscatter ratio can be described by the equation:

(9) $$p=\frac{\sigma_{HH}^{0}}{\sigma_{VV}^{0}}={\left(1-{\left(\frac{2\Theta }{\pi }\right)}^{\frac{1}{3\Gamma_{0}}}e^{-kh}\right)}^{2}$$

where *Γ_0_* is the Fresnel reflectivity coefficient at nadir (i.e., *Θ* = 0), which depends on the relative permittivity *ε_r_* of the surface material: $\Gamma_{0}={\left|\frac{1-\sqrt{\varepsilon_{r}}}{1+\sqrt{\varepsilon_{r}}}\right|}^{2}.$

Cross-polarized backscatter ratio can be calculated as

(10) $$q=\frac{\sigma_{VH}^{0}}{\sigma_{VV}^{0}}=0.23\sqrt{\Gamma_{0}}\left(1-e^{-kh}\right)$$

The magnitude of $\sigma_{VV}^{0}$ is described by the expression:

(11) $$\sigma_{VV}^{0}=gcos^{3}\Theta \left[\Gamma_{V}+\Gamma_{H}\right]/\sqrt{p}$$

where *p* is given by Equation (9), $g=0.7\left[1-exp\left(-0.65{\left(kh\right)}^{1.8}\right)\right]$, *Γ_V_* and *Γ_H_* are Fresnel reflectivity for vertically and horizontally polarized waves, respectively, at the incidence angle *Θ*. After calculating $\sigma_{VV}^{0}$ using Equation (11), the normalized RCS for other polarizations can be found from Equations (9) and (10).

From Equation (9) it follows that at small incidence angles the ratio of co-polarized signals *p* is close to one and decreases with increasing angle. The higher the roughness of the surface, the smaller the difference between normalized RCS at different polarizations. In addition, this ratio depends on the dielectric constant of the material. The cross-polarized ratio *q* is always much less than one and shows a stronger sensitivity to the surface roughness and a weaker dependence on the dielectric properties. In this study, we will test how this model matches the results of measuring signal backscattering at low THz frequencies.

### 3.3. Relative Permittivity of Surface Material

To use this empirical model, the dielectric properties of the surface material should be known. Sandpaper with grits 40, 80, 120, and 240, manufactured by Sealey Group (St Edmunds, UK), was chosen as a test surface (models PP232840, PP232880, WD2328120, and WD2328240, respectfully). Sandpaper grits are categorized according to the coarseness of the abrasive particles used. The sandpaper is composed of backing material (paper or woven fiber) with *ε_r_* = 2.0, covered with abrasive (aluminum oxide grains) with *ε_r_* = 9.7, and adhesive (resin) with *ε_r_* = 1.5–2.5 at 1 MHz frequency [35]. The value of effective dielectric permittivity can be obtained from the dielectric constants and volume fractions of constituents of the test material. However, such data is not available in the open literature.

To retrieve the dielectric constant of sandpaper over the complete low THz range, we used the Menlo Systems THz time-domain spectrometer TERA K15 Mark II in a quasi-optical configuration along with a material parameter extraction algorithm [36], similarly to [37]. This retrieval algorithm minimizes the difference between the measured (defined as ratio of the sample spectrum to the reference spectrum computed by Fourier transform of the corresponding waveforms) and theoretical complex transfer functions using the Nelder-Mead simplex algorithm, whereby the thickness- and frequency-dependent complex refractive index of the sample are extracted after numerical optimization.

To estimate the thickness of the sample, the system measures temporal separation between the leading pulse and its echo and between successive echoes within the sample. The soundness of the algorithm is validated by comparing the thickness output by the retrieval algorithm and that provided by the sandpaper manufacturer. 

**Figure 4:**
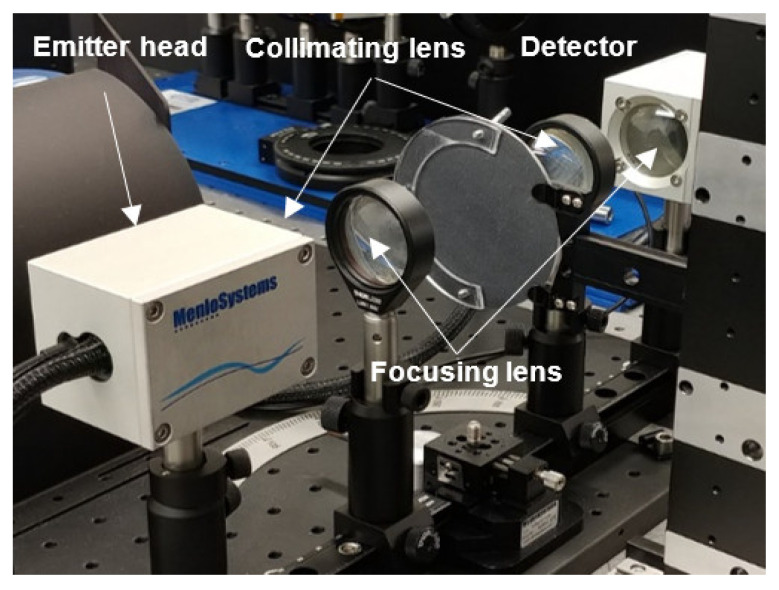
TDS system measurement set up.

To minimize the influence of scattering from the test surface in the retrieval method, we worked with a focused beam configuration using TPX planoconcave lenses [38] as shown in Figure 4. In such a configuration, the diffuse scattering, within ±10 deg. approximately [39,40], was collected owing to the relay lens in the detection side and contributed to the retrieval method.

A collimated configuration was also employed for large grit number (fine grit) sandpaper to check consistency of the data. An 80 mm diameter round sandpaper sample was placed in the sample holder at the focal plane of the optical system where the frequency-dependent beam-waist was estimated to be larger than 1 mm below 700 GHz [38,39]. To decrease systematic errors, a series of three independent reference and sample measurements was taken.

For the characterization of the average power of return from rough surface, effective parameters suffice. Assumed homogenization of the medium is a conventional approach for the modelling of microwave structures where an effective dielectric permittivity is assigned to the multilayered structure (which can be seen as weighted average of dielectric properties of individual layers such as abrasive, substrate, etc.). The simulation results presented below confirm this assumption as the dielectric permittivity used to generate analytical results agrees very well with measurements.

The average measured permittivity values of sandpaper as well as their standard deviations at low THz frequencies are shown in Table 1. Due to the coarse roughness of the 40-grit sandpaper sample, the retrieval algorithm only converged for one of the three runs. Hence, the absence of standard deviation in Table 1 for this case.

The loss tangent, which is the measure of signal loss due to the dissipation of electromagnetic energy in the sandpaper, can be defined as

(12) $$tan\;\delta =\varepsilon^{\prime \prime }/\varepsilon^{\prime }$$

where $\varepsilon^{\prime }$ and $\varepsilon^{\prime \prime }$ are the real and the imaginary components of permittivity. The measured values of *tan* δ are given in Table 1. In most cases, they are in the range of 0.20–0.30.

### 3.4. Measurement Setup

**Figure 5:**
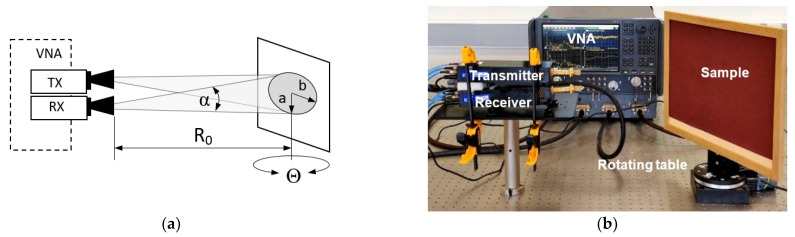
Experimental setup configuration (**a**) schematic setup, (**b**) actual setup.

For the backscattering experiments, we used the Keysight N5247B VNA available at the Terahertz measurement facility at the University of Birmingham, which can measure the full two-port scattering parameters in the frequency range from 10 MHz up to 1.1 THz using the frequency converter units [41]. The experimental setup is shown in Figure 5.

**Figure 6:**
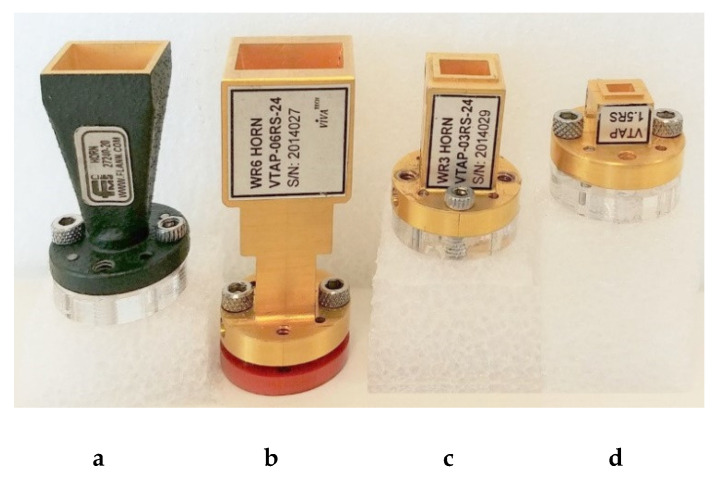
Antennas: (**a**) 79 GHz, (**b**) 150 GHz, (**c**) 300 GHz, and (**d**) 670 GHz.

The measuring system that corresponds to a quasi-monostatic radar with two closely spaced Tx and Rx antennas was stationary; only the test surface rotated and therefore the distance between the center of the illuminated area and antennas always remained the same. The rotation step was 5°, and the incidence angle varied from zero, when the antennas were perpendicular to the surface, to 80°, when they were almost parallel to the surface. Scattering coefficients were measured for co-polarized (vertical and horizontal polarization) and cross-polarized transmit and receive signals at frequencies of 79 GHz, 150 GHz, 300 GHz, and 670 GHz. Different polarizations were obtained by rotating either Tx or Rx modules. Specifications of the system and set-up parameters are provided in Table 2; the antennas are shown in Figure 6.

For 79 GHz measurement we chose 4 GHz bandwidth which is defined by the European frequency regulation [42] for automotive radars in the 79 GHz frequency band. At higher frequencies, the wider bandwidths can be readily achieved to improve range resolution. Therefore, to resolve extended targets/surfaces with the imaging radar the 16 GHz bandwidth has been used for 150 GHz, 300 GHz, and 670 GHz radar measurement and the bandwidth kept the same to compare the results. The sandpaper rms height was measured in [43], and they are shown in Table 3 together with measured total thickness with backing paper/fabric and calculated electromagnetic roughness. The parameters given in [43] should be considered approximate, since there are no strict standards for sandpaper and the roughness can vary from batch to batch within certain limits.

The sample with dimensions of 28 cm by 46 cm was fixed in a frame mounted on a rotating table at 30 cm from the antennas. This distance is sufficient, since the far field distance of the antennas, estimated by

(13) $$d_{F}=2D^{2}/\lambda$$

where *D* is the maximum linear dimension of the antenna (Table 2), did not exceed 20 cm.

From consideration of Figure 5a, it follows that the illuminated area is an ellipse with semi-minor and semi-major axes:

(14) $$a=R_{0}tan\frac{\alpha }{2}\;\mathrm{and}\;b=R_{0}tan\frac{\alpha }{2}\frac{cos\frac{\alpha }{2}}{cos\left(\frac{\alpha }{2}+\Theta \right)}$$

where *α* is the antenna beamwidth. Thus, in the case under consideration, only the semi-major axis *b* depends on the aspect angle to the sample, increasing with the increase of incidence angle, while the semi-minor axis *a* is 26 mm. At an incidence angle of 82° the sandpaper sample is not anymore beam filling and this defines the largest incidence angle for which measurements can be made. To remove diffraction and other possible reflections, the signal was range-gated from 15 cm to 45 cm.

Antenna beamwidth *α* = 10° (Table 2), and by approximating that $cos\left(\frac{\alpha }{2}\right)=cos\left(5{}^{\circ}\right)\approx 1,$ the area of the ellipse *A = πab* can be expressed as

(15) $$A\approx \frac{\pi R_{0}^{2}tan^{2}\frac{\alpha }{2}}{cos\left(\frac{\alpha }{2}+\Theta \right)}\;$$

Knowing RCS (Equation (8)) and *A* (Equation (15)), the normalized RCS can be calculated by Equation (3). The proposed experimental setup allows taking measurements more conveniently than traditional methods with a fixed sample and moving antennas [20], because it does not require the use of a rotating frame for antennas and modules and the distance remains unchanged at any incidence angle. However, this method is applicable only for lightweight samples that can be mounted vertically.

THz radiation penetration depth *Dp*, defined as the distance from the surface into the dielectric at which the traveling wave power drops to *e*^−1^ from its value at the surface, can be expressed as [32]:

(16) $$Dp=\frac{\lambda }{2\pi {\left(2\varepsilon^{\prime }\right)}^{1/2}}{\left\{{\left[1+{\left(\frac{\varepsilon^{\prime \prime }}{\varepsilon^{\prime }}\right)}^{2}\right]}^{1/2}-1\right\}}^{-1/2}$$

It should be noted that we are not applying the empirical model for scattering at 79 GHz. Therefore, considering Table 1 data and Equation (16), the maximum penetration depth is reached at 150 GHz and is in the range from 0.7 to 0.8 mm, which is more than the thickness of most sandpapers (see Table 3). At higher frequencies, the penetration does not exceed the thickness of the sandpaper. Thus, there is reflection from the surface and volume, and our experiment can be regarded as a special case of the scenario considered in [25].

## 4. Results and Discussion

In this section the results of normalized RCS measurement for sandpaper with grits of 40, 80, 120, and 240 at different low THz frequencies (79, 150, 300, 670 GHz) are presented and compared with the empirical model, which is described in Section 3.2. As can be seen from Table 3, the considered combinations of roughness and frequencies cover all possible ranges, from very smooth to rough surface. At a frequency of 79 GHz, all surfaces will be smooth or relatively smooth, and at a frequency of 670 GHz, most of them will be rough or relatively rough. The frequencies of 150 GHz and 300 GHz are intermediate options between these two extremes.

To avoid dependence of the result on any potential texture, which may happen during abrasive layer deposition or bends, we have changed orientation of the sample by rotating it within the same imaging plane. For convenience of measurement, each sample was rotated by a step angle and measured, clockwise and anticlockwise. In the paper we refer to different measurements of the same sample. In the figures below, normalized RCS are represented as smoothed curves using a third-degree polynomial approximation.

### 4.1. Normalized RCS in Vertical Polarization

**Figure 7:**
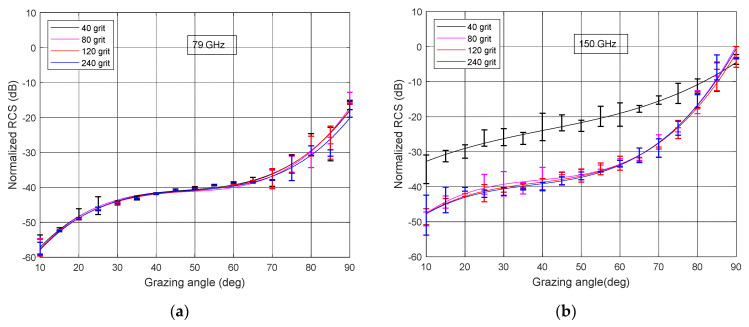

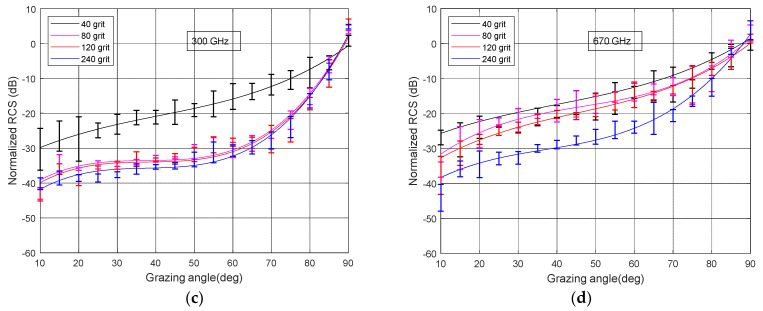
The normalized RCS $\sigma_{VV}^{0}$: (**a**) 79 GHz, (**b**) 150 GHz, (**c**) 300 GHz, and (**d**) 670 GHz.

As a reference point, we chose a radar with a center frequency of 79 GHz and 4 GHz bandwidth. The measured normalized RCS $\sigma_{VV}^{0}$ at 79 GHz is shown in Figure 7a for different sandpapers. The results were averaged over six or more measurements; the error bars show the obtained standard deviation.

As can be seen from Figure 7a, except for the final part of the graphs (grazing angle above 70°), the difference between $\sigma_{VV}^{0}$ of four sandpaper grits is within the accuracy of the measurement. At large grazing angles, the backscattering is highest because the geometry is close to the specular reflection direction. However, this is outside the range of angles of interest for automotive preemptive sensing. When the angle decreases, the backscattering signal decreases for all samples equally. Indeed, based on the values of sandpaper electromagnetic roughness (Table 3), all these samples are electrically “smooth”. Signal is mostly reflected away from the radar and the difference in backscattered signals would be insufficient for reliable classification.

The angular width of the specular reflection region depends on the antenna beamwidth. As the grazing angle decreases, the power *P_R_* of the backscattered signal drops down to almost noise level. In accordance with Equation (6), this power drop is a result of a decrease in the normalized RCS $\sigma^{0}$ (Equation (3)).

In Figure 7b, the result of normalized RCS $\sigma_{VV}^{0}$ measurement at 150 GHz is shown as a function of a grazing angle. The graph clearly shows the difference in reflection from 40-grit sandpaper compared to all other grits. Indeed, based on Table 3 in the first case we have a moderately rough surface with electromagnetic roughness 0.34 and in all other cases a smooth surface with electromagnetic roughness less than 0.2.

In accordance with the Fraunhofer criterion (Equation (1)), the diffuse reflection region for sandpaper with grit 40 occurs at a grazing angle above 35°. The normalized RCS of 40-grit sandpaper reduces at lower angles; however, it remains considerably higher than for smoother sandpapers.

Figure 7c depicts the result of $\sigma_{VV}^{0}$ measurement at 300 GHz as a function of a grazing angle for sandpapers with 40, 80, 120, and 240 grit. The reflection of a signal from 40-grit sandpaper is diffuse in almost the entire range of grazing angles, and in accordance with Equation (1) it has a specular reflection mechanism only below 17°.

We did not find any considerable differences in backscattering from sandpaper with grit 80 and 120. This is in some contradiction with the data in Table 3 where at a frequency of 300 GHz the electromagnetic roughness of sandpaper with grit 80 is 0.41 (mainly diffuse reflection), and sandpaper with grit 120 is 0.19 (mostly specular reflection). As we discussed above in the Section 3.2, the properties of the backscattered signals are determined not only by the roughness of the surface, but also by its dielectric constant.

The results for $\sigma_{VV}^{0}$ as a function of the grazing angle are shown in Figure 7d for 670 GHz. Graphs presented in Figure 7d significantly differ from the previous results (Figure 7a–c). Reflection from all types of sandpaper, except for sandpaper with grit 240, is predominantly diffuse. According to the Fraunhofer criterion, this reflection pattern is preserved, depending on the size of the grit, until the angle decreases to 15°–25°. With a further decrease in the grazing angle, the normalized RCS rapidly decreases. At the same time, an expected trend for return power is observed: the higher the roughness of the surface, the greater the power of the reflected signal. Reflection from 240-grit sandpaper is generally specular; the graph is characterized by a peak at high grazing angles with the width depending on the width of the antenna beam, and a rapid decrease at lower angles.

**Figure 8:**
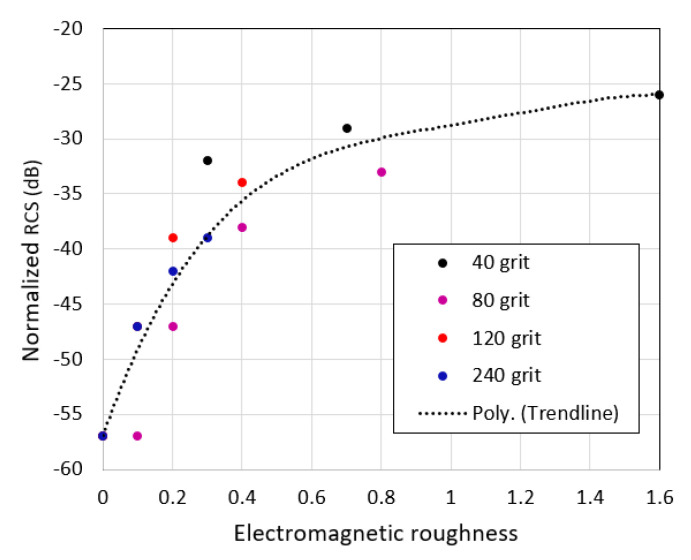
The normalized RCS $\sigma_{VV}^{0}$ as a function of electromagnetic roughness at a grazing angle of 10°.

Figure 8 shows discrete values of normalized RCS $\sigma_{VV}^{0}$ versus electromagnetic roughness (see Table 3) for different sandpapers at a grazing angle of 10°. The general trend for the normalized RCS to increase with increasing *kh* is clearly seen.

### 4.2. Measured and Calculated Normalized RCS

**Figure 9:**
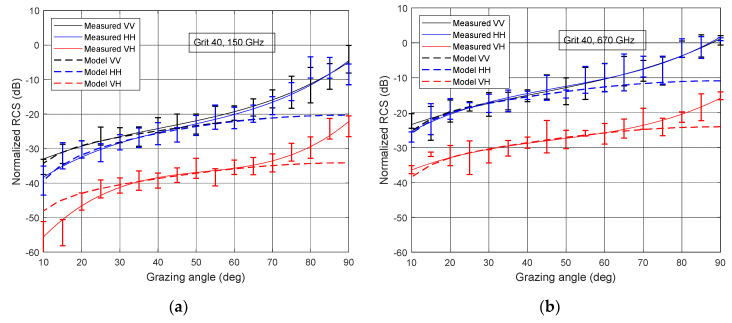

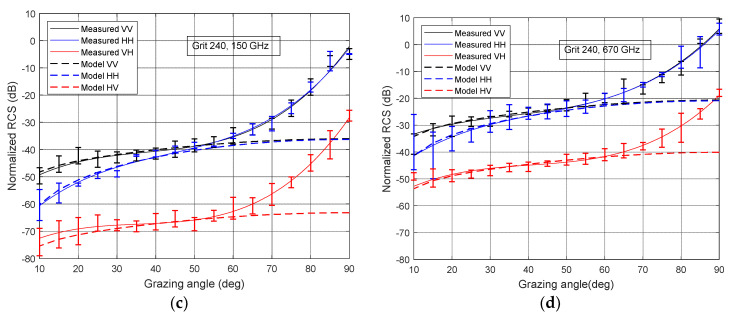
Measured and calculated normalized RCS: (**a**) grit 40 at 150 GHz, (**b**) grit 40 at 670 GHz, (**c**) grit 240 at 150 GHz, and (**d**) grit 240 at 670 GHz.

Figure 9 depicts the measured and calculated normalized RCS at different signal polarizations: $\sigma_{VV}^{0},$ $\sigma_{HH}^{0},$ and $\sigma_{HV}^{0},$ as a function of grazing angle increment. In this section, we restrict ourselves to considering reflection from sandpaper with two extreme values of roughness (grit 40 and grit 240) at two frequencies (150 GHz and 600 GHz), since the above examples illustrate well the general dependencies. The theoretical values are calculated using the empirical model explained in Section 3.2.

All considerations regarding the behavior of the graphs at different frequencies depending on the roughness of the surface, made during the discussion of Figure 7a–d, are valid for this case. As can be seen from the Figure 9a–d, at higher grazing angles the graphs for signals with horizontal and vertical polarization coincide within the measurement accuracy. It follows from expression Equation (8) that in all cases $\sigma_{HH}^{0}/\sigma_{VV}^{0}$ ≤ 1, and a noticeable difference between normalized RCS manifests itself with a decrease in the grazing angle. Calculations show that with an increase in the relative permittivity of the surface material, this difference also increases. In the example under consideration, *ε_r_* of the sandpapers at a frequency of 150 GHz lay in the range from 3.6 to 4.9 (Table 1). In the case of real road surfaces, the relative permittivity value can vary within a much wider range, for example, from 4.27 to 15.20 at 4.8 GHz [25]. We can expect that certain similar differences will exist in the low THz frequency range. This will make the difference between the curves $\sigma_{HH}^{0}$ and $\sigma_{VV}^{0}$ more noticeable and simplify the task of classifying surfaces.

As can be seen from Figure 9a–d, at grazing angles less than about 50°, the experimental results are in good agreement with normalized RCS calculated according to (9–11). This allows us to draw a conclusion about the applicability of the empirical scattering model, introduced in Section 3.3, for the low THz range of signals. It should be noted that the empirical model is valid only for cases of diffuse reflection and is not suitable for large grazing angles when the reflection mechanism changes to a specular one. This is the reason for the discrepancy between the experimental results and the model at large grazing angles. However, as already mentioned, we are interested in low grazing angles, and therefore this discrepancy does not seem significant.

The normalized RCS for cross-polarized signal $\sigma_{VH}^{0}\;\left(\sigma_{HV}^{0}\right)$ are significantly weaker than for co-polarized signal. In accordance with the empirical model, cross-polarized backscatter ratio *q* (Equation (10)) does not depend on the grazing angle. Indeed, in the case of 40-grit sandpaper, at a signal frequency of 150 GHz in the range of angles from 30° to 90°, the difference between the co-polarized and cross-polarized signal is about 16 dB (Figure 9a). At a signal frequency of 670 GHz, this difference was about 14 dB (Figure 9b). Similar dependences are observed for paper with grit 240 (Figure 9c,d).

Table 4 shows the ratio *q* measured in the range from 30° to 90°. As can be seen from the table, for each sample and frequency, this ratio varies within relatively small limits over the specified range of grazing angles.

As can be seen from Figure 9a–c, at low grazing angles, there was some difference between the model and the experiment. This may be the result of the backscattering return power approaching the noise floor of the instrument. This effect is especially noticeable when measuring cross-polarization returns $\sigma_{VH}^{0}\;\left(\sigma_{HV}^{0}\right)$, since the power of the backscattered cross-polarized signal becomes very small. In addition, the sandpaper sample is not ideally flat which affects the accuracy of the measurements for small grazing angle wherein the sample’s unevenness becomes more relevant.

### 4.3. Discussion

The difference in the power of the vertically polarized and horizontally polarized signal allows us to conclude about the dielectric parameters of the surfaces. In real road conditions, due to different dielectric permittivity, the difference between signals backscattered from different surfaces will be significantly larger than in the experiment.

For example, in [30] it is shown that at a frequency of 94 GHz the effective dielectric constant of road surfaces varies from 2.5 for dirty road to 4.2 for concrete. If we assume that the roughness of these surfaces is the same, then in accordance with (9–11), due to a different dielectric constant, the difference between normalized RCS of these surfaces at 45° will be 6.5 dB. For comparison, the measured at 150 GHz variations of *ε_r_* for sandpaper are in the range from 3.6 to 4.9 (see Table 1). This gives a difference in the normalized RCS due to *ε_r_* of only 2.6 dB. In particular, the analysis of backscattered signals can be used to determine surface moisture to detect ice and water on asphalt [19,20].

The backscattered cross-polarized signal also carries information about the properties of surfaces (roughness and dielectric constant); however, its low power at low grazing angles can make it difficult to extract and analyze the parameters of such a signal. The ability to use such information will depend on the practical implementation of the radar.

The absolute values of the reflected signals cannot serve as a reliable basis for surface identification, since they depend on the individual parameters of transmitter and receiver, their installation accuracy, pollution of the antenna radome (dirt, mud, snow, etc.). Therefore, the use of co-polarization and cross-polarization ratios, together with other signal characteristics, in our opinion, will give more reliable classification results.

The ability to distinguish surfaces gives good prospects for imaging radar in recognizing road markings, which may be important when developing autonomous cars [3]. As shown in [6,7], wide band 79 GHz automotive radar allows quite accurate image segmentation and classification of surfaces and obstacles which are the key technologies to provide valuable information for path planning in autonomous driving. From our results, it follows that increasing the frequency of the radar can potentially lead to more accurate discrimination of a larger number of surfaces.

Let us now consider the effect of surface clutter return on the recognition of road objects. Our results of measuring surface RCS allow us to draw important conclusions about the degree of this influence. As we report here, the normalized RCS decreases rapidly with decreasing grazing angle (increasing distance). Therefore, the power of the signal reflected from the road surface, which is proportional to $\sigma /R_{0}^{4}$ (Equation (4)), will decrease faster than the power of the signal reflected from the considered as point targets road objects which is proportional to $1/R_{0}^{4}.$ This leads to an increase in signal-to-clutter ratio. Let us say that in the case of 40 grit sandpaper at a frequency of 300 GHz, this ratio was 0 dB at an angle of 45°. Then, as follows from Figure 7c, with a decrease in the angle to 10°, due to a decrease in the normalized RCS, this ratio will increase to approximately 10 dB. Of course, we must consider that with a high-resolution radar, even road objects will be area scatterers so the simple $\;1/R_{0}^{4}$ rule does not really apply to them. However, the results we obtained give us confidence that such techniques might be feasible and with the range of tens hundreds of meters, reflection from the road surface will not impede the recognition of objects.

## 5. Conclusions

The aim of this work was to study the characteristics of low THz signal reflection from surfaces with different roughness in terms of applicability in automotive radars. We were interested in how the reflected signal can be used to classify surfaces and how much signal backscattering from the road can make it difficult to recognize road objects. We examined radar with frequencies: 79 GHz, 150 GHz, 300 GHz, and 670 GHz and measured the backscattering from sandpaper with grit 40, 80, 120, and 240. The resulting frequency and grit combinations cover all possible variants, from very smooth to very rough surfaces.

In general, three areas can be distinguished in the graphs of normalized RCS of co-polarized signal:

1. The initial section of the graph relates to the dominant specular reflection region, where the reflected signal is weak and rapidly decreases with increasing distance (decreasing grazing angle). The power of such a signal is likely to be insufficient for the classification of surfaces.
2. The diffuse reflection region, the extent of which is determined by the Fraunhofer criterion, where the reflected signal is strong enough for distinguishing surfaces with different roughness. This region may be almost absent when the signal is reflected from a smooth surface when the power of backscattering signal is low.
3. The third distinctive region where the level of the reflected signal is maximum, its angular range depends on the beamwidth of the antenna. The smoother the surface, the more prominent this region. With a very rough surface, this region is barely visible.

The use of a signal in the low THz range allows us to obtain diffuse reflection from road surfaces, which are smooth surfaces at the usual frequencies of automobile radars (24 GHz or 79 GHz).

The results of our experiments showed that the Fraunhofer criterion could serve as a sufficiently accurate guideline for determining the surface roughness characterization in the low THz range. The empirical scattering model, discussed in Section 3.2, showed good accuracy in diffuse reflection area in comparison with our measured result in the low THz range; it provides an important understanding of the features of radar signal backscattering. However, its use requires knowledge of the properties of surfaces, including roughness and the dielectric constant.

This work provides an insight into the effects of surface roughness on signal backscattering, which will play an important role in understanding the complex problem of signal reflection from actual road surfaces. The results obtained will allow us to select the features of the backscattered signal to effectively distinguish between road surfaces. The system we are developing will be an integral part of route planning systems for autonomous vehicles.

The novelty and importance of our results lie in experimental demonstration of an advantage of moving higher in frequency for the automotive Surface ID radar in terms of increased normalized RCS measured at various conditions and range of grazing angles, in confirmation of the applicability of the known models of signal backscattering to the region of low THz frequencies, and in substantiating the possibility of surface identification by analyzing the parameters of polarized backscattered signal.

Our further plan is to study the signal reflection from asphalt, gravel, sand, grass, etc. at low THz frequencies, paying particular attention to the peculiarities of reflection from coatings formed by weather conditions (water, snow, ice).

## Figures and Tables

**Table 1:** Sandpaper relative permittivity.

Frequency		Sandpaper Grit			
		40	80	120	240
150 GHz	*ε_r_*	3.6	3.6	4.4	4.9
	*σ_εr_*	−	0.1	0.3	0.2
	*tan* δ	0.25	0.25	0.29	0.24
300 GHz	*ε_r_*	3.4	3.4	3.9	4.6
	*σ_εr_*	−	0.1	0.5	0.2
	*tan* δ	0.27	0.28	0.30	0.22
670 GHz	*ε_r_*	2.9	3.5	3.1	4.3
	*σ_εr_*	−	0.1	0.3	0.2
	*tan* δ	0.25	0.22	0.49	0.20

**Table 2:** Parameters of the measurement system.

	Frequency, GHz			
	79	150	300	670
Frequency band, GHz	77–81	142–158	282–298	656–672
Wavelength, mm	3.8	2.0	1.0	0.4
Sweep bandwidth, GHz	4	16	16	16
Transmitted power, dBm	6	−6	−9	−25
Antenna azimuth beamwidth (−3 dB)	10°	10°	10°	10°
Antenna elevation beamwidth (−3 dB)	10°	10°	10°	10°
Antenna gain, dBi	20	24	25	20
Antenna aperture dimensions, mm	11 × 15	17 × 18	6 × 8	3 × 4
Far field range, mm	76	202	128	71
Range resolution, mm	37.5	9.4	9.4	9.4

**Table 3:** Sandpaper Parameters.

Grit	Thickness, mm	Surface rms, mm	Electromagnetic Roughness *kh*			
			79 GHz	150 GHz	300 GHz	670 GHz
40	1.25	0.11	0.18	0.34	0.69	1.55
80	0.46	0.06	0.10	0.19	0.38	0.84
120	0.43	0.03	0.05	0.09	0.19	0.42
240	0.28	0.01	0.02	0.03	0.06	0.14

**Table 4:** Cross-polarized backscatter ratio *q*, dB.

Grazing Angle	30°	60°	90°
Grit 40, 150 GHz	−16	−16	−17
Grit 40, 670 GHz	−13	−14	−14
Grit 240, 150 GHz	−26	−26	−26
Grit 240, 670 GHz	−19	−20	−22

## Data Availability

The data that support the findings of this study are available from the corresponding author, A.B. upon reasonable request.
